# Time Lag and Communication in Changing Unpopular Norms

**DOI:** 10.1371/journal.pone.0124715

**Published:** 2015-04-16

**Authors:** Klarita Gërxhani, Jeroen Bruggeman

**Affiliations:** University of Amsterdam, Department of Sociology, Amsterdam, Netherlands; Middlesex University London, UNITED KINGDOM

## Abstract

Humans often coordinate their social lives through norms. When a large majority of people are dissatisfied with an existing norm, it seems obvious that they will change it. Often, however, this does not occur. We investigate how a time lag between individual support of a norm change and the change itself hinders such change, related to the critical mass of supporters needed to effectuate the change, and the (im)possibility of communicating about it. To isolate these factors, we utilize a laboratory experiment. As predicted, we find unambiguous effects of time lag on precluding norm change; a higher threshold for a critical mass does so as well. Communication facilitates choosing superior norms but it does not necessarily lead to norm change when the uncertainty on whether there will be a norm change in the future is high. Communication seems to help coordination on actions at the present but not the future. Hence, the uncertainty driven by time lag makes individuals choose the status quo, here the unpopular norm.

## Introduction

When people meet, they can stabilize their mutual expectations through shared norms: informally sanctioned rules that prescribe or proscribe certain behavior. For reducing cognitive load and facilitating coordination on certain outcomes of social encounters while discouraging others, the emergence and maintenance of social norms have often been explained by the advantages they provide to those who adhere to them [1–3]. There is, however, substantial evidence of norms that harm those who comply. Examples are norms on footbinding in China [4], female circumcision in Africa [4], bribery and corruption in various cultures [5], the mass suicide of Jim Jones’ sect when it was threatened in Guyana [6], and the easy access to and possession of guns in the United States. Although a majority of individuals may disapprove of such norms when asked individually, the pertaining group collectively conforms to them. What explains this conformity to unpopular norms and why do people not succeed in implementing change? In this paper, we aim to provide answers to these questions.

Various explanations for adherence to unpopular norms have been proposed. These include (i) a lack of or inaccurate information in uncertain situations, leading people to copy the behavior of others, even if doing so goes against their own beliefs and preferences, which is referred to as herd behavior [7, 8]; (ii) a misinterpretation of others’ beliefs about how to behave in a certain situation, leading to a suppression of one’s own beliefs and a tendency to copy the majority, which is labeled pluralistic ignorance [5, 9]; and (iii) not only adhering to the norm but also enforcing it to signal dedication to the norm despite privately questioning it, which is known as false enforcement [6, 10]. The first two explanations imply that the provision of more accurate information would suffice to diminish the commitment to unpopular norms. The false enforcement explanation stresses the importance of social pressures to adhere to a norm. In this view, the provision and dissemination of information are seen as a starting point to affect changes to unpopular norms.

In some cases, information provision has contributed to the overthrow of unpopular norms, such as the end of footbinding in China [4]. In many other cases, unpopular norms persist (see the remaining examples above). These cases suggest that, in actuality, a change that benefits all (or most) may not occur even when awareness of improvement is raised. What impedes the change of unpopular social norms? We focus on an under-researched aspect of norm change: the time lag between individuals’ decisions to change a norm and the outcome of that decision. A time lag may affect the chances of norm change along two channels, time-lag uncertainty and time-lag discounting. First, the longer the temporal distance between a decision and its outcome, the higher the uncertainty about what the outcome will be and the (possibly unstable) commitment of others to the alternative norm. In particular, other things being equal, a longer time lag makes it less likely that current decisions to change a norm will eventually lead to an outcome where this change is realized. One reason is that commitments and many other things can change between the original decision and the final outcome. In attempting to explain the endurance of female circumcision in Africa, Knight and Ensminger [11] argue: “We believe that the exceptional persistence of this norm is due in large part to the long time lag between a change in the norm (at the age of eight when circumcision is carried out) and evidence of the costs associated with change (at marriage, which may be at sixteen to twenty years of age).” As this example shows, deviations are only punished after a long time lag (i.e. non-marriageable daughters many years later), not when the decision of (no) norm change is being made (i.e. circumcise one’s own daughter or not). This contrasts with cases without time lag and where punishment of deviant behavior plays an important role in maintaining the current norm [12]. Second, the longer the time lag, the more a future outcome is discounted in the present [13]. When applying this second aspect to the valuation of a difference between two alternative norms, the magnitude of this difference decreases in the future. Thus, if people must commit now to obtain a better norm in the future, they are less likely to do so than if the outcome of the superior norm were experienced immediately because the size of the improvement appears smaller from a temporal distance. This discussion leads to the following general *proposition*: the longer the time lag between individual decisions to support a norm change and the change itself, the smaller the chance that the individual will act to change a norm. We argue that this decrease is caused by (1) a lower likelihood that the norm will eventually change; and (2) a smaller value difference between the current and alternative norm, due to discounting.

Below, we will use these arguments to derive specific hypotheses related to our research design, which is a laboratory experiment. Our experimental design allows us to isolate our main variable of interest, time lag. The measurement of individual and collective behavior with the controlled variation of one variable at a time also helps us to better understand the interactions between time lag and other variables with respect to norm change. We investigate two such variables in particular: a critical mass and communication. A disadvantage of the experimental method, however, is that it excludes specific historical conditions and long-term socialization. We acknowledge that outside the laboratory, effective norms are often domain specific and, hence, history dependent. Our study of several key factors that potentially affect the dynamics of social norms is therefore only part of the story. In our view, however, a proper understanding of such dynamics requires an accurate comprehension of its underlying factors.

## Critical Mass and Communication

While focusing on the effect of time lag, we also consider that individuals’ decisions are partly based on their expectations of others’ decisions about whether to conform to a norm. To change a norm, individuals will take into account the number of others who agree upon the change. What counts as enough others varies across individuals and situations, and in a given situation there will be a distribution of individuals’ crossover points [14, 15]. If the number of initiators reaches a critical mass, the norm change becomes self-sustaining. The critical mass then changes individual’s expectations such that it “converts the slightest sign of plurality into an overwhelming majority” [16]. In a game-theoretic analysis of the coordination problems involved in footbinding in China and female circumcision in Africa, Mackie [4] argues that in both cases a majority was stuck in an inferior equilibrium. He theorizes that setting a norm change in motion (toward a superior equilibrium) would be possible if a small group of individuals who did not favor the existing norm began to express dissatisfaction and to organize, such as by forming an association. Subsequently, a critical mass needs to be reached at which a sufficient number of individuals share their dissatisfaction with the existing norm. Thus, we predict that the lower is the threshold for the critical mass, the higher will be the chance that the norm will change.

On the basis of historical data on footbinding, Mackie showed that a critical mass is easier reached when sufficient information is transmitted about the drawbacks of the current norm and the advantages of an alternative norm. Communication about current and alternative norms may play an important role as it influences expectations. With respect to decision outcomes, however, the literature on group communication features two opposing theories. One involves free, or relational, spaces where norm reformers can meet in the absence of proponents of the status quo to enhance mutual solidarity and trust and to coordinate their actions [17, 18]. In a free space, good ideas can be nourished, and a social movement can self-organize and subsequently topple the proponents of an inferior norm to implement a superior one. Many experimental studies have indeed shown a positive effect of communication on cooperation [19]. This effect is reached in the short time span of a laboratory experiment, which suggests that communication can influence the direction of behavior even in a confined laboratory environment. An opposing theory on groupthink [20] predicts that group communication homogenizes an initially diverse range of opinions in a rather thoughtless manner, often accidentally excluding the best idea [21]. Thus, it may diminish the opportunity to improve group members’ fates. Once group members have synchronized their opinions, subsequent interactions enhance confidence in the collective opinion irrespective of whether this opinion reduces or increases group members’ payoffs [20, 21]. Taken together, these two strands of literature imply that communication in social interactions can help a group to implement a superior norm or, in contrast, enhances the entrenchment of the inferior one or has a neutral effect at best. In sum, if communication works out as in a free space, it increases the likelihood of choosing the superior norm, whereas if it results in groupthink, it decreases the likelihood of choosing the superior norm.

## Materials and Methods

We first discuss the game we use as a basis for our experiment, in which we isolate our main variables of interest.

### The game

Groups of n players must choose one of two alternative norms, A or B, that are characterized by their payoffs. Because only one norm can hold for all players in a group, it is a coordination game. We set the payoffs such that we can interpret B as the current norm and A as the alternative norm, even though the players in the experiment are unaware of our interpretation and have no shared history with traditions and new norms (see S1 Text). If enough players choose A, with respect to a threshold that we vary in the experiment, the group norm becomes A; otherwise, it remains B. Adhering to B represents the baseline option, for which we give a payoff of 100 points. If a player is the only one joining the alternative norm, we give her a low payoff of 20 points (which represents a decrease in the costs of joining an unpopular norm, e.g., a health risk imposed on a circumcised daughter), such that her net result is lower than conforming to the ruling norm B.

In actuality, thresholds for critical masses are difficult to predict [14]. In our experiment, in contrast, we keep this threshold under control, so that we can isolate its effect on the likelihood of norm change. Rather than requiring a large majority for one norm or the other, we vary the threshold of the critical mass that determines the norm. If a fraction α∊[0, 1] or more of the population (i.e., experimental group) chooses the alternative norm A, then it applies to everyone. Consequently, the payoffs for those who choose A are higher than for those who maintain the current norm B. If, in contrast, a lower fraction than α opts for A, then the norm does not change. For example, if α = 0.5, then the norm is determined by the majority’s choice; the payoffs are shown in Table 1.

**Table 1:** Payoffs and critical mass.

	At least α choose A	Less than α choose A
A (alternative norm)	120	20
B (current norm)	0	100

Payoffs of player under consideration, given threshold α for everyone’s choices (critical mass).

Similar to Mackie’s [4] ranked equilibria game, the game in Table 1 constitutes a coordination game among n players, with two pure strategy equilibria: everyone maintains the current norm, or everyone joins the alternative norm. Of these two outcomes, the second is the efficient equilibrium because everyone is better off when no one circumcises, bribes, or purchases guns than when everyone does.

In line with the discussion of the two aspects of time lag (uncertainty and discounting), we introduce time lag in two ways. In our game, we model time-lag uncertainty by introducing a probability p that the norm will change when the threshold for change is reached: in case of certainty, p = 1; with uncertainty, p < 1. Hence, p describes the probability that the payoffs in the column “at least α choose A” (Table 1) will be realized. Consistent with the theoretical discussion above, uncertainty corresponds to the belief that even if enough people change their behavior now, there is a probability 1-p that the change of current norm B will not be realized in the future due to the temporal distance and many things that can change in between. In this way, choosing the alternative norm A constitutes participating in a lottery with probabilities p and 1-p. A change in p has two effects. On the one hand, it changes the expected value of group coordination on the alternative norm A. Given that the (fixed) payoff to current norm B remains unchanged (because no time lag is involved in keeping the existing norm), this uncertainty changes the relative attractiveness of A versus B. This uncertainty-induced change in relative attractiveness is captured by the expected value of the lottery involved in choosing A. The second effect of a change in p is a change in the risk involved in the lottery associated with choosing A. For example, aside from the expected value, a lottery with p = 0.9 is less risky than a lottery with p = 0.5. The risk-effect disappears, however, for risk-neutral individuals. In this paper, we abstract from this risk-effect of changes in uncertainty. It would require introducing randomized payoffs in the experiment (which would make it necessary to take into account participants’ risk aversion). Our focus is on the first effect, the change in relative attractiveness. Hence, we use the expected payoff that follows for a given p. An analysis of the risk-effect is left for future research.

Our emphasis on expected value implies that changes in probability can be captured in the payoffs that are shown to the subjects; see Table 2. Note that if there is no uncertainty about the outcome, and the subjects believe that the current norm will not change no matter what they do (p = 0), the two columns in Table 2 are identical to each other. At the other extreme, when there is no uncertainty because the current norm will change with certainty if a sufficient number of people act accordingly (p = 1), the payoffs are identical to those in Table 1. For intermediate values of p, the subject sees her payoffs as a weighted average of the two columns from Table 1.

**Table 2:** Payoffs, critical mass and uncertainty.

	At least α choose A	Less than α choose A
A (alternative norm)	20(1-p) + 120p = 100p+20	20
B (current norm)	100(1-p) = 100-100p	100

Payoffs of player under consideration, given threshold α (critical mass) and probability p (time-lag uncertainty). Maintaining the current norm is a dominant strategy if 100p+20 < 100-100p, i.e. if p < 0.4.

The second aspect of time lag, time-lag discounting, is implemented by introducing a real time lag of one week between the experimental session and the moment when the payoff can be collected. We set p = 1, implying that in theory there is no time-lag uncertainty. We implement the real time lag only in the case of choosing the alternative norm A, in line with Mackie’s theory that discusses abolition of footbinding and circumcision as alternatives (to be realized in the future) to current norms. In our experiment this means that for some outcomes, the participants had to return to the laboratory after one week to collect their earnings. Note that many come to campus regularly anyway, to follow classes or to use university facilities. We therefore consider costs of collecting payoffs to be minimal. Importantly, although collecting the payoff was delayed by one week, participants were informed of the outcome at the end of the experiment. By removing this outcome uncertainty together with the time-lag uncertainty (p = 1), we can isolate the effect of time-lag discounting. We cannot however exclude the possibility that subjects experienced some psychological uncertainty. Some examples of the latter are whether they will be paid at all if payoff is postponed by one week (i.e. experimenters might change their mind), or subjects may fear of being sick or occupied when having to collect the money. Although one week might seem a short period of time, for discounting the largest effect is predicted when comparing now with a time point in the near future, whereas comparisons between now and time points further away lead to a decreasing marginal effect of discounting [13]. The payoff table used for the real time lag treatment is presented in the design section below.

### Subjects and procedures

The game was implemented in the spring of 2012 at the CREED Laboratory of the University of Amsterdam. More than 2000 potential participants in the CREED subject pool received an invitation to participate, and participation was on a first-come, first-served basis. We recruited 195 participants, ranging in age from 18 to 34 years (mean = 22.5 years). The participants were mainly undergraduate students.

CREED is a renowned institute for experimental economic research and adheres to the standards set in experimental economics. These standards include: participation on a voluntary basis; participants are paid in cash, where earnings are dependent on decisions made; no deception of participants is permitted; and, all data are collected on an anonymous basis and the choices are not linked to specific individuals. The collection, storage, protection, retention, and destruction of all data comply with national and EU regulations.

All participation is on a strictly voluntary basis. Participants are recruited from the CREED subject pool, where they themselves can register if they wish to participate. Registration involves consent with the procedures at CREED. This recruitment procedure has been approved for all experiments at CREED by the internal review board (IRB) of the Faculty of Economics and Business of the University of Amsterdam. The IRB, chaired by prof. dr. J. Sonnemans, has granted standard approval to all experiments that adhere to the rules set by the Center for Research in Experimental Economics and political Decision making (CREED). No specific approval for specific experiments, such as ours, is required.

Each subject participated in one of eight experimental sessions consisting of nine rounds in a row. Throughout all sessions, the participants played in anonymous groups of five (n = 5) and were informed that group composition was randomly reshuffled after each round. In the experimental room, each participant sat at a computer in a cubicle that prevented visual contact with others, and before participants entered the room, we instructed them not to communicate with each other. Before each session began, the participants were introduced to the same instructions via the computer; see S1 Text for a translation of the instructions. The options of committing to the current norm or selecting the alternative one were formulated neutrally as a choice between options B and A, respectively. The participants were asked whether they understood the instructions and were instructed to raise questions if they did not. At the end of the sessions, the participants completed a short questionnaire about demographic characteristics and the main motives for their decisions in the experiment. The sessions lasted approximately 45 minutes, and the participants’ payoffs were based on their decisions (see Tables 3 and 4). Earnings in the experiment were in points. Subjects were informed that they would be paid 0.7 eurocents for each point they earned. On average, they earned €17, including a €7 show-up fee (a default value at the CREED laboratory).

**Table 3:** Critical mass and time-lag uncertainty.

Scenario 1	3 or more participants choose A	fewer than 3 choose A
You choose A	70	20
You choose B	50	100
**Scenario 2**	3 or more participants choose A	fewer than 3 choose A
You choose A	110	20
You choose B	10	100
**Scenario 3**	at least 1 participant chooses A	nobody chooses A
You choose A	70	not applicable
You choose B	50	100
**Scenario 4**	at least 1 participant chooses A	nobody chooses A
You choose A	110	not applicable
You choose B	10	100

From top to bottom: scenario’s 1–4. Scenario 1: High threshold (α = 3/5) and high uncertainty (p = 0.5). Scenario 2: High threshold (α = 3/5) and low uncertainty (p = 0.9). Scenario 3: Low threshold (α = 1/5) and high uncertainty (p = 0.5). Scenario 4: Low threshold (α = 1/5) and low uncertainty (p = 0.9).

**Table 4:** Critical mass and time-lag discounting.

Scenario 5	3 or more participants choose A	fewer than 3 choose A
You choose A	960 one week later	160 immediately
You choose B	0	800 immediately
**Scenario 6**	at least 1 participant chooses A	nobody chooses A
You choose A	960 one week later	not applicable
You choose B	0	800 immediately

Scenario 5: High threshold (α = 3/5) and discounting. The payoffs are larger so that the total payoff from scenarios 1–2 (over eight rounds) and scenario 5 (round nine) are similar.

Scenario 6: Low threshold (α = 1/5) and discounting. The payoffs are larger so that the total payoff from scenarios 3–4 (over eight rounds) and scenario 6 (round nine) are similar.

### Design

The design combines within-subject and between-subject treatment variation. In particular, the treatment variables concerning time-lag uncertainty (captured by the probability p) and critical mass (captured by α) were varied within subjects, meaning that each subject was confronted with multiple values of p and α, as described below. The treatment variable communication was varied between subjects, meaning that each subject was either in a session where communication was allowed or in one where it was not possible. The experiment consisted of two parts, elaborated below.

#### Part 1: Critical mass and time-lag uncertainty

In this part of the experiment, we examined the participants’ behavior with respect to variations in the threshold for the critical mass and the level of time-lag uncertainty. To these ends, we implemented a high versus low threshold for the critical mass needed to choose the alternative norm A, for which we set α = 3/5 and α = 1/5 (see S1 Text), respectively, and high and low levels of uncertainty, p = 0.5 and p = 0.9, respectively. The resulting 2x2 factorial led to four different payoff scenarios. In Table 3, we present the scenarios as they were shown to the participants. All participants were confronted with each scenario twice in random order, amounting to a total of eight rounds. These were played in part 1, with variations in critical mass and time-lag uncertainty. The ninth round was played in part 2 (described below), where variations in critical mass were combined with real time lag (i.e. time-lag discounting).

#### Part 2: Critical mass and time-lag discounting

In part 2, the two different thresholds of reaching a critical mass were combined with the real time lag. This combination resulted in two payoff scenarios, presented in Table 4. Each participant was confronted with one of these two scenarios, in the ninth round. The payoffs for these two scenarios (5 and 6) are larger so that they remain comparable, in terms of payoffs, to the other scenarios that were played over the first eight rounds in part 1.

To test the effect of communication, we conducted sessions with and without a communication possibility for scenarios 1–6. When there was communication, each participant could interact with the other four members of the group before making a choice. Communication was established through a chat window on the computer (the experimental free space), which all members of the same group could use to communicate (anonymously) for one minute before choosing option A or B.

### Hypotheses

Based on this experimental design, we formulate four hypotheses on the behavior to be expected in our experiments. These pertain to the main proposition about time lag and the additional predictions on critical mass and communication.

First, the proposition that an increased time lag reduces the likelihood that individuals will act to change a norm yields:

- *Hypothesis 1* (uncertainty): Participants will choose option A more often when p = 0.9 than when p = 0.5.
- *Hypothesis 2* (discounting): Participants will choose option A more often when p = 0.9 and payoff is immediate than when p = 1 and payoff is delayed by one week (see S1 Text).

Our prediction that lowering the threshold for critical mass increases the likelihood that individuals will act to change a norm yields:

- *Hypothesis 3*: Participants will choose option A more often when α = 1/5 than when α = 3/5.

Finally, the two contrasting predictions on the effect of communication yield:

- *Hypothesis 4a* (free space): In the presence of chat, participants will choose more often the option with the highest payoff than without chat.
- *Hypothesis 4b* (groupthink): In the presence of chat, participants will choose less often the option with the highest payoff than without chat.

## Results

We present the results in the order of the hypotheses, where italicized *p* referring to the chance that the hypothesis is false needs to be distinguished from the p we use to indicate time-lag uncertainty. For the experiment, subjects participated in groups of n = 5 that were reshuffled after each round. The matching groups of subjects from which the experimental groups were drawn contained 10 or 15 people. We had a total of 12 matching groups with 10 subjects and 5 matching groups with 15 subjects. Average A-choices did not differ significantly across these two matching group sizes for any of the scenarios considered (Mann-Whitney, all *p* > 0.195). Because subjects’ decisions and chats may have affected others within the same matching group in later rounds, we treat the matching groups as the independent units of observation when conducting our analyses (unless indicated otherwise). Table 5 gives an overview of the sample sizes.

**Table 5:** Sample sizes of subjects and matching groups.

	α = 1/5, no-chat	α = 1/5, chat	α = 3/5, no-chat	α = 3/5, chat
# subjects	50	45	45	55
# matching groups	4 (2 of 10; 2 of 15)	4 (3 of 10; 1 of 15)	4 (3 of 10; 1 of 15)	5 (4 of 10, 1 of 15)

Since most treatments were run within subjects, and only communication was varied between subjects, the table presents the sample sizes of subjects and matching groups in the chat and no-chat treatments. Treatments vary within subjects only for decisions in round nine. All 17 matching groups did all p (i.e. time-lag uncertainty) and α (i.e. critical mass) variations.

A first observation is that when subjects encounter the same scenario a second time (in the first eight rounds), they support the alternative norm A more frequently than the first time around, which points at a learning effect. To avoid confounds caused by learning effects, the analysis that follows allows for learning and is based on decisions made by subjects the second time.

**Fig 1:**
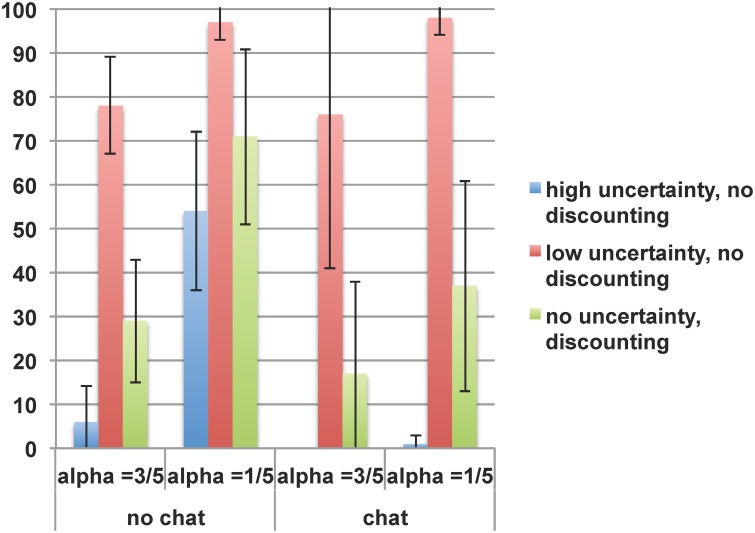
Time-lag discounting and uncertainty, with critical mass and communication. Error bars show one standard deviation above and below the mean. In correspondence with using matching groups as the unit of observation, averages and standard deviations are calculated with respect to the means per matching group.

To test our first two hypotheses on the effect of time-lag, Fig 1 compares choices for the alternative norm A in the two uncertainty scenarios and the actual delay scenarios. It clearly shows that when time-lag uncertainty is low, far more people choose A (bars in the middle) than in all scenarios with high uncertainty (bars at the left). Using two-sided Wilcoxon test (N = 17), the difference between low and high uncertainty is significant at *p* < 0.001, which supports Hypothesis 1: time lag implemented as uncertainty decreases the likelihood of social norm change. A remarkable result is that when the threshold for critical mass is low (α = 1/5), but time-lag uncertainty is high and communication is not allowed, many choose the alternative norm A, even though B is the option with the highest payoff (c.f. Table 3). This is probably the case because in addition to high uncertainty about the norm eventually changing, subjects cannot communicate, thus are not able to coordinate their decisions at the present. Moreover, since in the low threshold condition a single choice for norm A is sufficient for it to be the group’s norm, it is chosen more often. Under the same critical mass and uncertainty conditions *with* communication, which allows for coordination, subjects win each other over to choose norm B, which is the most profitable choice after all.

Our complementary approach to the time-lag uncertainty effect is implementing a real time lag, in the experiment lasting one week, which involves the psychology of discounting. As described in the design section, this case involves in theory no uncertainty (p = 1). To test Hypothesis 2, i.e. to compare the effects of time-lag uncertainty and time-lag discounting, we use the case with near certainty (p = 0.9) and no real time lag as a benchmark. Using the Wilcoxon test (N = 17), this hypothesis is supported at *p* < 0.001, which suggests that most subjects do indeed discount future benefits and choose to stay with the current norm. Fig 1 shows that compared to this benchmark the effect size of the time-lag discounting is smaller than that of higher time-lag uncertainty (p = 0.5). Although both effects point significantly in the same direction, subjects are the least inclined to choose the alternative norm A when time-lag uncertainty is high (p = 0.5), and the most inclined when the time-lag uncertainty is low (p = 0.9). In the case of time-lag discounting their choices fall in between. This is remarkable given that in the case of time-lag discounting, the uncertainty level is the lowest possible (p = 1). Therefore, even though time-lag uncertainty seems to have the strongest effect in impeding norm change, time-lag discounting is also influential, and this is the case beyond uncertainty reasons that subjects might have.

**Fig 2:**
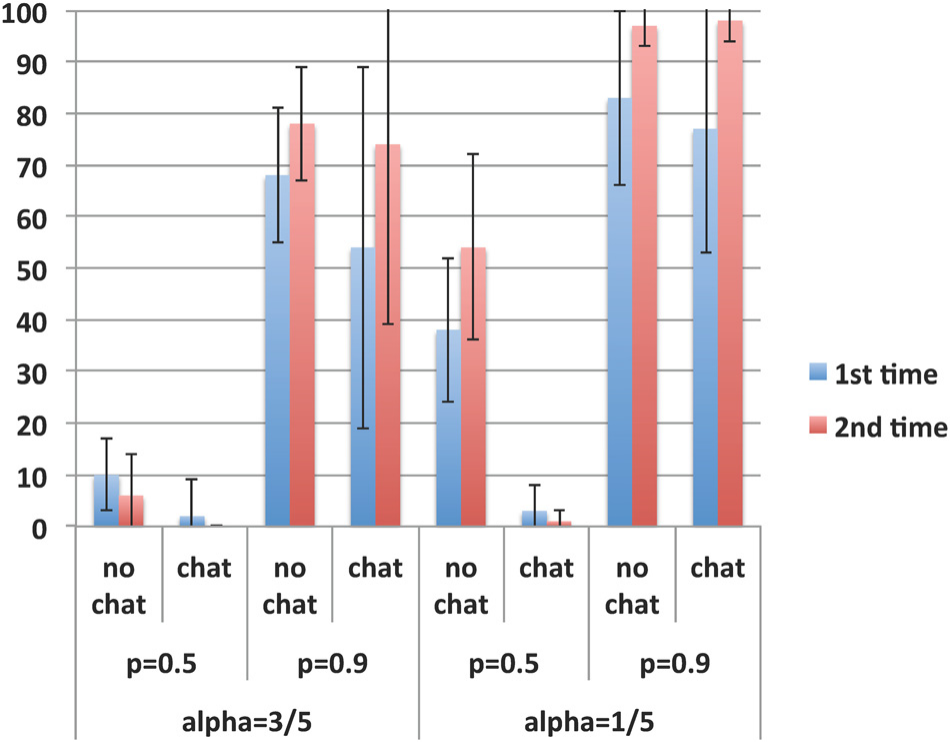
Critical mass, time-lag uncertainty, and communication. Bars show the percentage of choices for option A in each scenario (first eight rounds). Time-lag uncertainty and critical mass scenarios were varied within subjects; each scenario was played a first time (blue, on the left) and a second time (red, on the right). The possibility to communicate via chat was varied between subjects. High and low thresholds for the critical mass are indicated by α = 3/5 and α = 1/5, respectively. Error bars show one standard deviation above and below the mean. In correspondence with using matching groups as the unit of observation, averages and standard deviations are calculated with respect to the means per matching group.

We now turn our focus to the third hypothesis on the effect of critical mass. Fig 2 gives an overview of all choices made across the different scenarios in the first eight rounds, expressed in percentages in favor of the alternative norm A.

Fig 2 indicates a strong effect of the threshold for the critical mass. In almost all comparisons for specific communication/uncertainty combinations, option A is chosen more often when the threshold is low (α = 1/5) than when it is high (α = 3/5). To test these differences, we use averages for each of the N = 17 matching groups. Aggregated across all treatments, Wilcoxon signed rank tests on paired observations strongly support Hypothesis 3, with *p* = 0.001. Breaking this down for communication and time-lag uncertainty yields: Without communication,

- High uncertainty (p = 0.5; N = 8), *p* = 0.012: in favor of Hypothesis 3.
- Low uncertainty (p = 0.9; N = 8), *p* = 0.018: in favor of Hypothesis 3.

With communication,

- High uncertainty (p = 0.5; N = 9), *p* = 0.317. This finding is less surprising than it might seem: in the scenarios under comparison (scenarios 1 and 3, Table 3), the current norm B is the superior one as it yields the highest payoff. Under high uncertainty on whether there will be a norm change in the future, communication helps coordinate the choices made at the present, leading group members to choose the efficient norm B, irrespective of whether the threshold is high or low. This explains the non-significant difference between the two scenarios.
- Low uncertainty (p = 0.9; N = 9), *p* = 0.078: in favor of Hypothesis 3.

These results show that within the overall support for Hypothesis 3, communication reduces the effect of the threshold for the critical mass under high time-lag uncertainty.

Our final hypotheses are on the effect of communication, which consists of a series of messages sent by the participants. Each message could be read by all group members. Discursive support for one or the other option was usually expressed at the beginning of the chat only, constituting approximately half of the messages, followed (or interspersed) by small talk, both on and off topic. This support can be seen as promises for choices to be made after the chat; at the group level, promises and choices were consistent. Fig 2 shows both cases where communication through chat increases the fraction of choices for the alternative norm A and cases where option A is chosen less often after chat. In aggregate, 58.9% of the subjects choose norm A without chat, whereas only 43.2% do so with the chat option. To test these differences we use the two-sided Mann-Whitney test, since communication was varied between treatments. Aggregated across all threshold/uncertainty combinations, the difference is supported at *p* = 0.001 (N = 17, U = 67.5). Hence, in aggregate our results indicate that communication does not facilitate norm change.

To better understand this aggregate finding, we analyze all combinations of uncertainty and critical mass separately, which give the following results: High threshold for critical mass (α = 3/5),

- High uncertainty (p = 0.5; N = 17): A is chosen 6.3% without chat, and 0% with chat. The Mann-Whitney test yields a nearly significant difference (*p* = 0.093, U = 54).
- Low uncertainty (p = 0.9, N = 17): A is chosen 78.3% without chat, and 74.1% with chat. The difference is very small and insignificant (*p* = 0.888, U = 34.5).

Low threshold for critical mass (α = 1/5),

- High uncertainty (p = 0.5, N = 17): A is chosen 53.7% without chat and only 0.7% with chat, which is a very significant difference (*p* < 0.001, U = 72).
- Low uncertainty (p = 0.9, N = 17): A is chosen 97.1% without chat and 98.1% with chat, which is a small and insignificant difference (*p* = 0.673, U = 31).

As discussed above, communication seems to help group members make the efficient choice, which is confirmed by the content of the chat.

We conclude that the positive effect of communication is supported, in line with Hypothesis 4a, whereas we did not find much evidence for Hypothesis 4b. This is especially the case when uncertainty on whether there will be a norm change in the future is high. Since maintaining the current norm B is the most efficient choice, communication allows group members to coordinate on the choice made at the present by opting out on the alternative norm A. When uncertainty about the norm eventually changing is low, choosing the alternative norm A is already recognized as the best option as almost everyone chooses A, thus leaving not much room for communication to make any further difference. This detailed analysis provides some explanation for the aggregate finding of communication not facilitating norm change, as communication turns out to matter only when time-lag uncertainty about the norm eventually changing is high. The way it matters, as the content of the chat pointed out, is by pushing the group members towards the efficient outcome at the present, which is maintaining the current norm.

In addition to these findings it is interesting to examine systematically whether the effect of time lag is robust in the presence of communication. First, we compare high and low time-lag uncertainty for treatments with and without communication.

Without communication,

- High critical mass (α = 3/5, N = 8): difference between high and low uncertainty significant at *p* = 0.012.
- Low critical mass (α = 1/5, N = 8): difference between high and low uncertainty significant at *p* = 0.012.

With communication,

- High critical mass (α = 3/5, N = 9): difference between high and low uncertainty significant at *p* = 0.010.
- Low critical mass (α = 1/5, N = 9): difference between high and low uncertainty significant at *p* = 0.006.

Taken together, these results show that even in the presence of communication, a time lag implemented as uncertainty decreases the number of choices for the alternative norm A.

Second, we compare the real one-week time-lag treatment and certainty (p = 1) to the case with near certainty (p = 0.9) and no real time lag. It is important to emphasize that we have fewer matching groups than in earlier comparisons, because subjects participated in only one time-lag discounting treatment (either high or low critical mass). We compare choices in the treatment a matching group participated in to their choices in the near certainty treatment with the same critical mass.

Without communication:

- High critical mass (α = 3/5, N = 4): difference between immediate and delayed payoff significant at *p* = 0.066.
- Low critical mass (α = 1/5, N = 4): difference between immediate and delayed payoff nearly significant at 10%, with *p* = 0.108.

With communication:

- High critical mass (α = 3/5, N = 5): difference between immediate and delayed payoff significant at *p* = 0.068.
- Low critical mass (α = 1/5, N = 9): difference between immediate and delayed payoff significant at *p* = 0.066.

These results show that communication does not take away the effect of time lag, even when implemented as actual delay. The modest significance level might be due to the low number of matching groups.

To enhance the robustness of our findings, we also perform a multivariate regression analysis; see Table 6. Here we do not use choices made in the time-lag discounting treatment, however, because these choices differ along too many dimensions from the choices made in all other treatments. We use probit regressions to explain the likelihood of choosing the alternative norm A. To correct for statistical dependencies within matching groups, we use robust standard errors clustered at the level of matching groups. This analysis reconfirms our results above and shows that, other things being equal,

- Subjects facing a low threshold for the critical mass are 33.4% more likely to choose the alternative norm A (Hypothesis 3).
- Subjects in communication sessions are 28.8% less likely to choose the alternative norm A.
- Subjects facing low uncertainty are 72.2% more likely to choose the alternative norm A (Hypothesis 1).

**Table 6:** Multivariate analysis of the likelihood of the alternative norm being chosen.

	Marginal effect	z-value
Communication possible	-0.288	5.68***
Low time-lag uncertainty	0.722	16.24***
Low threshold	0.334	6.30***
Second time scenario faced	0.165	4.87***
Subject’s age/100	-0.954	2.47**
Subject is woman	-0.016	0.64
Subject has paid job	0.086	1.96**
Subject studies economics or business	-0.004	0.10

n = 1560 total choices on all available variables, 17 matching groups.

***significant at 1% level,

**significant at 5% level.

Other findings are that the second time a subject faces a scenario, (s)he is 16.4% more likely to choose the alternative norm, which confirms the learning effect mentioned earlier. Furthermore, the tendency to choose the alternative norm decreases with age; students with a paid job are 8.6% more likely to choose the alternative norm than students without; and, gender and majoring in economics/business have no significant effect on choosing the alternative norm.

We also performed the regressions separately for sessions with and without communication (see Table 7). The most important finding is that even in the presence of communication via chat, time-lag uncertainty decreases the likelihood of social norm change. The rest of the results are similar to the ones reported in Table 6.

**Table 7:** Multivariate analysis, separately for sessions with and without communication.

	No communication		Communication	
	Marginal effect	z-value	Marginal effect	z-value
Low time-lag uncertainty	0.610	12.21***	0.771	13.20***
Low critical mass	0.398	8.13***	0.201	2.76***
Second time scenario faced	0.136	3.29***	0.169	3.47***
Subject’s age/100	–0.668	1.28	–1.043	1.79*
Subject is woman	–0.010	0.26	–0.039	1.81*
Subject has paid job	0.094	1.42	0.053	1.05
Subject studies economics or business	0.041	0.72	–0.067	1.62
	n = 760	8 matching groups	n = 800	9 matching groups

***significant at 1% level,

**significant at 5% level.

## Discussion and Conclusion

Humans coordinate part of their social lives through norms, and when they are dissatisfied with a current norm, they opt for an alternative one—or so it might seem. However, there are various hurdles along the way. Even when an alternative norm and its advantages are known by those involved, an unpopular (current) norm is not always changed. From an evolutionary point of view, this outcome seems paradoxical because contrary to genes, culture, which incorporates norms, enables relatively flexible adaptation to changing circumstances [22]. Our research question asked why, in many circumstances, people do not succeed in substituting an alternative norm for an unpopular one. In evolutionary terms, our exemplary unpopular norms (female circumcision, footbinding, corruption, gun access, and mass suicide) decrease people's fitness (i.e., survival chance, offspring, or both).

Of the various factors that may be at play, our primary focus was on time lag, which was intertwined with critical mass and communication. To distinguish these three effects and to exclude confounding factors beyond our control, we chose an experimental method. This method triangulates with Mackie's [4] study that is based on game theory and the analysis of historical data. Our results are unambiguous for the effect of a time lag: a temporal distance between individual decisions to support a norm change and the change itself decreases the likelihood that an unpopular norm will change. This holds both when time lag is implemented as uncertainty about the norm eventually changing in the future, and when it is implemented as a real-time lag of discounting the future. Importantly, as the effect of time lag in case of the latter is observed for a one-week time lag only, it may well be larger in actual situations with longer time lags. Our findings apply to an existing unpopular norm and can be generalized to situations in which norms are lacking altogether and new ones can emerge and be maintained. A case in point is the depletion of irreplaceable natural resources [23], which Hardin [24] described as a “tragedy of the commons”. In this case, a new norm (or a bundle of norms, [25]) that proscribes depletion would increase the survival of future generations. As a side effect, such a norm would strongly incentivize the current generation to search for alternative resources or technologies. Our study suggests that the time lag between individuals’ decisions to commit to new norms to protect natural resources and the outcome of such a decision (which is experienced much later by future generations) hinders the emergence and maintenance of such protective norms. A higher threshold for the critical mass required to join a new norm is an additional obstacle.

Although communication may help individuals organize and achieve collective willingness to move toward an alternative norm, the coordination of new expectations must be arranged to ensure that the alternative norm will be applied at a future time point [11]. Knowing that the majority will commit to the alternative norm in the present may lead to a change of the current norm if everyone is aware that the outcome of that change will be experienced immediately rather than after a time lag. For example, in the case of corruption norms, Bicchieri and Fukui [5] argue that more information dissemination on the true beliefs of businesspeople (i.e., condemning corruption) will lead to the change of such an unpopular norm. We add to this argument that such change may occur because, in that example, the consequences for businesspeople to operate in a ‘clean’ and fair business environment are close in time to their decision to commit to the alternative norm. If the majority shares a belief in condemning corruption, that majority is likely to readily experience a fair business environment. As we have shown, this is more difficult to achieve when the time lag is long. Communication seems to facilitate coordination in choosing superior norms at the present, but it does not necessarily lead to norm change, in particular when the uncertainty of the norm eventually changing in the future is high.

In our experiment, we investigated endogenous factors that hinder or facilitate norm change. Outside the experiment, there is also an important exogenous factor: competition between groups. In sports, for example, teams have equipment and clothing, training and a strategy that apply to all group members. Teams with superior material, training and strategies outcompete other teams. Eventually, the losers imitate the winners, whose norms and other cultural elements then diffuse throughout the population of teams, replacing inferior norms in their wake. In business, firms that are better aligned with their environment (e.g. due to norms that appeal to their audience) outcompete other firms [26]. Some firms imitate the innovations of successful firms, and those with inferior norms go bankrupt. Darwinian selection at the group level does not mean that group members go extinct, but if external competitive pressure is high enough due to highly opinionated consumers or scarce resources, firms with misaligned norms go out of business [26]. Their members then have to find new jobs in other firms, where they are re-socialized in different norms, and the current norms vanish with the firms that cultivated them. Group selection also plays an important role in the evolution of cooperation [24]. It might seem that this cannot hold true because of migration between groups and cultural flexibility; groups would be too unstable for selection to apply [27]. However, inter-group migration is perfectly consistent with group selection as long as group cultures continue to differ from each other [28]. Our results contribute to this debate by showing that although some cultural elements are flexible, several factors account for the inertia of norms such that groups continue to differ from each other in the long run.

### Ethics Statement

This paper includes the collection and analysis of personal data based on the voluntary cooperation of human beings in the laboratory of CREED at the University of Amsterdam. CREED is a renowned institute for experimental economic research and adheres to the standards set in experimental economics. These standards include: participation on a voluntary basis; participants are paid in cash, where earnings are dependent on decisions made; no deception of participants is permitted; and, all data are collected on an anonymous basis and the choices are not linked to specific individuals. The collection, storage, protection, retention, and destruction of all data comply with national and EU regulations.

All participation is on a strictly voluntary basis. Participants are recruited from the CREED subject pool, where they themselves can register if they wish to participate. Registration involves consent with the procedures at CREED. This recruitment procedure has been approved for all experiments at CREED by the internal review board (IRB) of the Faculty of Economics and Business of the University of Amsterdam. The IRB, chaired by prof. dr. J. Sonnemans, has granted standard approval to all experiments that adhere to the rules set by the Center for Research in Experimental Economics and political Decision making (CREED). No specific approval for specific experiments, such as ours, is required.

## Supporting Information

S1 Text Methodological notes and experimental instructions. (PDF)

## References

[1] Yamagishi T. The Provision of a Sanctioning System as a Public Good. J Pers Soc Psychol. 1986; 51:110–16.

[2] Horne C. Sociological Explanations of the Emergence of Norms In: Hechter M, Opp KD, editors. Social Norms. New York: Russell Sage; 2001 pp. 3–34.

[3] Elster J. Explaining Social Behavior. Cambridge: Cambridge University Press; 2007.

[4] Mackie G. Ending Footbinding and Infibulation. Am Sociol Rev. 1996; 61: 999–1017.

[5] Bicchieri C, Fukui Y. The Great Illusion: Ignorance, Informational Cascades and the Persistence of Unpopular Norms. Bus Ethics Q. 1999; 9: 127–155.

[6] Willer R, Kuwabara K, Macy MW. The False Enforcement of Unpopular Norms. Am J Sociol. 2009; 115: 451–490. 20614762 10.1086/599250

[7] Banerjee A. A Simple Model of Herd Behavior. Q J Econ. 1992; 107: 797–817.

[8] Bikhchandani S, Hirshleifer D, Welch I. A Theory of Fads, Fashion, Custom, and Cultural Change as Informational Cascades. J Pol Econ. 1992; 100: 992–1026.

[9] Miller DT, McFarland C. When Social Comparison Goes Awry: The Case of Pluralistic Ignorance In: Suls J, Wills TA, editors. Social Comparison: Contemporary Theory and Research. Hillsdale, NJ: Erlbaum; 1991 pp. 287–313.

[10] Centola D, Willer R, Macy MW. The Emperor’s Dilemma: A Computational Model of Self-Enforcing Norms. Am J Sociol. 2005; 110: 1009–1040.

[11] Knight J, Ensminger J. Conflict over changing social norms In: Brinton MC, Nee V, editors. The New Institutionalism. Stanford, Stanford University Press; 1998 pp. 105–126.

[12] Fehr E, Fischbacher R. Third-Party Punishment and Social Norms. Evol Hum Behav. 2004; 25: 63–87.

[13] Berns GS, Laibson D, Loewenstein G. Intertemporal choice – toward an integrative framework. Trends Cogn Sci. 2007; 11: 482–488. 17980645 10.1016/j.tics.2007.08.011

[14] Granovetter M. Threshold models of collective behavior. Am J Sociol. 1978; 83: 1420–1443.

[15] Easley D, Kleinberg J. Networks, Crowds, and Markets. Cambridge: Cambridge University Press; 2010.

[16] Schelling TC. The Strategy of Conflict. Cambridge: MA Harvard University Press; 1960.

[17] Polletta F. Free spaces in collective action. Theor Soc. 1999; 28: 1–38.

[18] Kellogg KC. Operating rooms: relational spaces and micro-institutional changes in surgery. Am J Sociol. 2009; 115: 657–711. 20503740 10.1086/603535

[19] Ledyard JO. Public goods: A survey of experimental research In Kagel J, Roth AE, editors. Handbook of Experimental Economics. Princeton NJ, Princeton University Press; 1996 pp. 111–194.

[20] Janis IL. 2nd ed Groupthink: Psychological Studies of Policy Decisions and Fiascoes. Boston: Houghton Mifflin; 1982.

[21] Lorenz J, Rauhut H, Schweitzer F, Helbing D. How social influence can undermine the wisdom of crowd effect. P Natl Acad Sci USA. 2011; 108: 9020–9025. 10.1073/pnas.1008636108 21576485 PMC3107299

[22] Richerson PJ, Boyd R. Not by Genes Alone. Chicago: The University of Chicago Press; 2005.

[23] Grantham J. Be persuasive. Be brave. Be arrested (if necessary). Nature. 2012; 491: 303 10.1038/491303a 23151541

[24] Hardin G. The tragedy of the commons. Science. 1968; 162: 1243–1248. 5699198

[25] Ostrom E. Beyond Markets and States. Am Econ Rev. 2010; 100: 641–672.

[26] Hannan H, Pólos L, Carroll G. Logics of Organization Theory: Audiences, Codes and Ecologies. Princeton, NJ: Princeton University Press; 2007.

[27] Rogers A R. Group selection by selective emigration: the effects of migration and kin structure. *Am Nat*. 1990; 135: 398 *–* 413.

[28] Boyd R, Richerson PJ. Transmission coupling mechanisms: cultural group selection. Philos T Roy Soc B. 2010; 365: 3787–3795. 10.1098/rstb.2010.0046 21041204 PMC2981912

