# Supplementary material for: Time Lag and Communication in Changing Unpopular Norms
Source: PLoS One. 2015 Apr 16;10(4):e0124715. doi: 10.1371/journal.pone.0124715 (PMC4399934; doi:10.1371/journal.pone.0124715)
Supplement: S1 Text — (PDF) [file pone.0124715.s001.pdf]

# Time Lag and Communication in Changing Unpopular Norms

**Klarita Gërxhani and Jeroen Bruggeman**

# S1 Text.

# Methodological notes

The rationale for setting the payoffs in the experiment such that we can interpret B as the current norm and A as the alternative norm is that we are interested in the effect of time lag in the most elementary case, of two alternative norms where socialization does not play a role. Since we expect a time lag to decrease the chances of norm change, allowing for a shared history where one of the two norms is based on a long standing tradition would increase the difficulty of changing the norm even further.

In the instructions (see below), the threshold for the critical mass refers to the number of members needed to maintain the current norm B (3 and 5, respectively). In the design, this threshold, $\alpha$, denotes the fraction of group members needed for the group norm to become the alternative norm A. These two representations describe precisely the same conditions. To facilitate the discussion of our experimental setting and its relation to real world examples, we prefer to use the latter in the paper. We do however acknowledge that there could be a framing effect of the former. It is a priori difficult to know which of the two is more natural. Since our aim is not to study framing effects, we decided to choose one of the two for the experiment, i.e. the latter.

When discussing the parameters of the game, we argue that there is some uncertainty related to delayed payoffs. This makes a comparison to the p=0.9 treatment our preferred choice to test hypothesis 2. Alternatively, we could have organized a separate treatment where p=1 and payoff is immediate. The actual differences in payoff numbers between p =

0.9 and p = 1 are small, however, hence we do not expect this choice to affect our overall conclusions on time lag.

# Experimental Instructions

Here we present an English translation of the Dutch instructions to the subjects in the experiment. Capitals and paragraphs are exactly as in the original. *Italics indicate either places where alternative texts were used for other treatments, or a clarifying remark for the reader*.

WELCOME

You are about to participate in an experiment. In this experiment, you can earn money. The amount of money you earn will depend on your own decisions and on the decisions of other participants. Additionally, you will receive a show-up fee of 7 euros.

In the experiment, payoffs are computed in terms of points. Your earnings in points will be paid to you in euros at the end of the experiment. This will be done privately, one participant at the time. The exchange rate in this experiment is 70 eurocent for 100 points.

It is important that you understand the instructions well. We therefore ask you to read them carefully. The instructions contain examples with numbers. The numbers are only used as illustration; they do not have any particular meaning for the experiment itself.

These instructions consist of various pages. You can page forward by clicking with your mouse on “next page”.

INTRODUCTION

The experiment consists of two parts. You will now receive the instructions for part 1. You will receive the instructions for part 2 after part 1 is finished.

PART 1

Part 1 consists of eight rounds.

In each round you will be allocated to a group together with four other participants. This allocation is random and anonymous. You don’t know who else is in your group and the others don’t know whether they are in the same group as you. In each of the eight rounds you will be allocated to a new group. You will never be in the same group of five in two consecutive rounds.

CHOOSING

In each round you have to choose between two options indicated by “A” and “B.” You can earn money with your choice.

How much you earn depends on how many members of your group (including yourself) choose A or B. In other words, there is a certain threshold for the group that influences how much you earn with your choice.

Here is an example. In this example, the threshold is 2. Your payoff for choosing A, if 2 or fewer members of your group choose B, is 10 points, and for choosing B is 20 points. Your payoff when the group is above the threshold is 40 points for A and 5 points for B. All this is displayed in the following table.

| Threshold 2 | 2 or fewer than 2 participants choose B | More than 2 participants choose B |
|---|---|---|
| You choose A | 10 | 40 |
| You choose B | 20 | 5 |

The thresholds and payoffs for A and B differ in the eight rounds. For each round, you will be shown the pertaining values.

After each round you'll be able to see how much you have earned in that round and how much you have earned in total, including earnings of previous rounds.

This is the end of these instructions. If you have finished reading them, click the button "ready" (at the bottom of this screen). We begin the experiment when everybody is ready.

COMMUNICATION
*[only visible to those participants who were assigned randomly to a communication treatment]*

In each round, before choosing A or B, you can communicate for one minute with other members of your group. You can do this via a chat screen. After this one minute, the chat screen closes and you can make your choice for A or B.

You can type your text in the space made available for the chat. The text will become visible to other group members by pressing the "enter" key.

The identity numbers of other players you see on the screen are randomly assigned each round, and are therefore unrelated to the same persons from previous rounds. Communication other than through the chat screen is prohibited. We ask you to conceal your true identity (name and/or number of your computer).

This is the end of these instructions. If you have finished reading them, click the button "ready" (at the bottom of this screen). We begin the experiment when everybody is ready.

PART 2

Everybody has finished part 1. We continue with part 2.

Part 2 consists of one round. You are assigned randomly to a group of five participants.

Again, you can choose between options A and B. Here also there is a threshold. The threshold is 2 *[4]*.

This part is different because the payment of your earnings will be postponed for about one week after today if your group does not exceed the threshold. You will be told at the end of the experiment whether this is the case. Those who have to receive their payment after one week will at the end get a receipt to collect their earnings on [*a date one week after the experiment*] between 15:30 and 16:30 at room nr. 6.35, building E. If you cannot collect your money at the given time slot, you can make a different appointment after the experiment.

*[Those who play scenario 5 get to see the following: threshold 2]*

If in your group 2 or fewer members (including yourself) choose B, then your payoff is 0 if you choose B and 960 points (to receive next week) if you choose A. If in your group more than 2 members (including yourself) choose B, you will be paid immediately after the experiment. You will then receive 800 if you choose B and 160 if you choose A. These options are summarized in the table below.

| Threshold 2 | 2 or fewer than 2 members choose B | More than 2 choose B |
|---|---|---|
| You choose A | 960 next week | 160 now |
| You choose B | 0 | 800 now |

*[Those who play scenario 6 get to see the following: threshold 4]*

If in your group 5 members (including yourself) choose B, you get your payment today. You receive 800 points if you choose B. Because this is possible only if everybody chooses B, the option of choosing A is not applicable. Of course, you or others can choose A. In that case the threshold of 4 is not surpassed and you will be paid next week. If the group stays below this threshold and you choose A, you get 960 points, but 0 points if you choose B. These options are summarized in the table below.

| Threshold 4 | 4 or fewer than 4 members choose B | 5 choose B |
|---|---|---|
| You choose A | 960 next week | not applicable |
| You choose B | 0 | 800 now |

This is the end of these instructions. If you have finished reading them, click the button “ready” (at the bottom of this screen). We begin the experiment when everybody is ready.

COMMUNICATION
*[only visible to those participants who were assigned randomly to a communication treatment]*

Here again, before choosing A or B, you can communicate for one minute with other members of your group. After this one minute, you can make your choice for A or B.

Communication other than through the chat screen is prohibited. We ask you to conceal your true identity (name and/or number of your computer).

This is the end of these instructions. If you have finished reading them, click the button "ready" (at the bottom of this screen). We begin the experiment when everybody is ready.

END

This brings you to the end of this experiment.

We kindly ask you to fill out a brief questionnaire.

If you are finished answering the questions, you can click the button "ready." Then please wait quietly until you are called to collect your payment.

EXIT QUESTIONS

We would like to ask you a few questions:

What is your age: ……

What is your gender: … male… female

Starting year of your study: ……

Your major (none if you are not a student): …..

Do you have a paid job for more than 2 days a week … yes … no

If in part 2 (immediate versus delayed payment) you chose A or B, can you tell us your reasons for doing so?

You chose A because:

…………………………………………………………………..

You chose B because:

………………………………………………………………….

Your answers will remain confidential.

This is the end of the questionnaire. We kindly ask you to remain quiet and seated until everyone is finished with the questionnaire.

Thank you very much for your cooperation!
